# Structure and magnetism of two chair-shaped hexanuclear dysprosium(iii) complexes exhibiting slow magnetic relaxation†

**DOI:** 10.1039/c7ra11378a

**Published:** 2018-01-03

**Authors:** Zi-Yuan Liu, Hua-Hong Zou, Rong Wang, Man-Sheng Chen, Fu-Pei Liang

**Affiliations:** State Key Laboratory for Chemistry and Molecular Engineering of Medicinal Resources, School of Chemistry & Pharmacy of Guangxi Normal University Guilin 541004 P. R. China gxnuchem@foxmail.com; Guangxi Key Laboratory of Electrochemical and Magnetochemical Functional Materials, College of Chemistry and Bioengineering, Guilin University of Technology Guilin 541004 P. R. China fliangoffice@yahoo.com

## Abstract

Two novel hexanuclear Dy^III^ complexes with polyhydroxy Schiff-base ligands, [Dy_6_(L^1^)_4_(μ_3_-OH)_4_(MeOH)_4_]Cl_2_·2MeOH·2MeCN (1) and [Dy_6_(HL^2^)_2_(μ_3_-OH)_2_(μ_3_-OCH_3_)_2_(piv)_10_(MeOH)_2_] (2) (H_3_L^1^ = *N*,*N*′-bis(3-methoxysalicylidene)(propylene-2-ol)-1,3-diamine, H_3_L^2^ = 2,3-dihydroxypropylimino)methyl)-6-methoxyphenol, piv = pivalate), have been prepared under solvothermal conditions and structurally characterized by single-crystal X-ray diffraction, elemental analyses, thermal analyses, and IR spectroscopy. Each of the hexanuclear complexes is constructed with Dy_3_ triangular motifs as building blocks, and the six Dy^III^ ions are arranged in a chair-shaped conformation. Variable-temperature magnetic susceptibility measurements in the temperature range of 2–300 K indicate dominant ferromagnetic exchange interactions between the Dy^III^ ions in the complexes. Both complexes exhibit slow magnetic relaxation behavior.

## Abstract 2

We report two novel chair-shaped hexanuclear Dy^III^ complexes with different polyhydroxy Schiff-base ligands. Both complexes are constructed with Dy_3_ triangular motifs as building blocks and exhibit slow magnetic relaxation behavior at low temperature.

## Graphical abstract

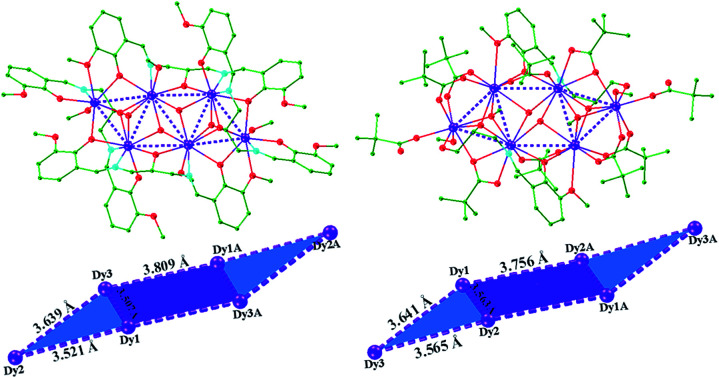

## Introduction

The synthesis of new single-molecule magnets (SMMs) containing lanthanide metals continues to grow because of their fascinating magnetic behaviours and potential applications in many fields such as high-density information storage, quantum computing, molecular spintronics *etc.*^1–6^ Most of the 4f-based SMMs reported in the literature are derived from Dy^III^ ion due to its unquenched orbital angular momentum and large intrinsic magnetoanisotropy.^7–11^ In the past decade, a number of mono- and polynuclear Dy^III^ complexes have been synthesized and characterized,^12^ and many of them have been demonstrated to exhibit remarkable properties of single-molecule magnets. For example, mononuclear Dy^III^ complexes with pentagonal bipyramidal coordination geometry featuring strong axial ligand field and weak equatorial donors exhibit very high effective energy barriers for magnetic relaxation.^13^ A dinuclear Dy^III^ complex bridged by an N_2_^3−^ radical ligand was observed to show magnetic hysteresis up to 8.3 K.^14^ A tetranuclear Dy^III^ alkoxide cage complex and a square-based pyramid iso-propoxide-bridged pentanuclear Dy^III^ complex have been reported to possess large thermal energy barrier for the reversal of magnetization.^1*b*,15^

Among the various Dy^III^-based SMMs reported, the Dy_3_ triangular molecules have been found to be a magnetically interesting system. This system has an essentially nonmagnetic spin ground state, but exhibits SMM behavior of thermally populated excited states, as well as toroidal magnetic moments which are useful in molecule-based multiferroics.^16^ Stimulated by the fascinating magnetic behaviors of these intriguing Dy_3_ triangles, research on the utilization of theirs as building blocks to construct larger SMMs with enhanced magnetic properties has attracted much attention. It was reported that linking of two such highly anisotropic Dy_3_ triangles in different forms led to the creation of hexanuclear dysprosium SMMs,^17^ of which enhanced slow magnetic relaxation was observed,^17*b*^ and enhanced toroidal magnetisms were obtained by fine-tuning the arrangements of the Dy^III^ ions or by modifying the local ligand-field around the Dy^III^ ions.^17*d*,*e*^ Combination of two independent Dy_3_ SMM-building blocks by a paramagnetic [Dy(μ_2_-CH_3_O)_2_Dy]^4+^ linker afforded an octanuclear Dy^III^ complexes with SMM behavior inherited from its Dy_3_ precursor.^18^ An even larger cluster of decanuclear Dy^III^ based on the peculiar Dy_3_ triangles was obtained by incorporating two sets of vertex-sharing Dy_3_ triangular motifs.^19^ These successful examples represent a promising strategy for preparation of novel Dy-based SMMs by using highly anisotropic Dy_3_ triangles as building blocks.

**Scheme 1:**
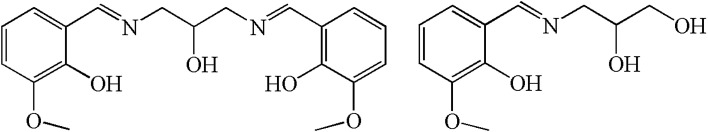
Schiff-base ligands of H_3_L^1^ (left) and H_3_L^2^ (right).

Herein, we report the synthesis, structure, and magnetic properties of two analogous hexanuclear Dy^III^ complexes, [Dy_6_(L^1^)_4_(μ_3_-OH)_4_(MeOH)_4_]Cl_2_·2MeOH·2MeCN (1) and [Dy_6_(HL^2^)_2_(μ_3_-OH)_2_(μ_3_-OCH_3_)_2_(piv)_10_(MeOH)_2_] (2), (H_3_L^1^ = *N*,*N*′-bis(3-methoxysalicylidene)(propylene-2-ol)-1,3-diamine, H_3_L^2^ = 2,3-dihydroxypropylimino)methyl)-6-methoxyphenol, and piv = pivalate, Scheme 1). The hexanuclear complexes can be viewed as being constructed with Dy_3_ triangular motifs as building blocks. The six Dy^III^ ions are arranged in a chair-shaped conformation that has never been shown for the hexanuclear lanthanide complexes. In addition, intramolecular ferromagnetic interactions are observed, which is rarely reported for the lanthanide SMMs.^17*a*,20^ Magnetic investigations indicate that both of complexes exhibit single-molecule magnets behavior.

## Experimental section

### Materials and methods

Metal salts and solvents were purchased from commercially available and used directly without further purification in the preparation of the free ligands and complexes. The Schiff-base ligands H_3_L^1^ and H_3_L^2^ were prepared as previously described.^21^ IR spectra were recorded in the range of 4000–400 cm^−1^ on Perkin-Elmer Spectrum Two FT/IR spectrometer using a KBr pellet. Elemental analysis (C, H, N) was performed on a Elementar Micro cube CHN elemental analyzer. The thermal analysis was performed on Labsys Evo TG-DTG/DSC. The crushed single-crystal sample was heated up to 1000 °C in N_2_ (99.99%) at a heating rate of 10 °C min^−1^. Magnetic susceptibility measurements were performed in the temperature range of 2–300 K, using a Quantum Design MPMS SQUID-XL-5 magnetometer equipped with a 5 T magnet. The diamagnetic corrections for these complexes were estimated using Pascal's constants, and magnetic data were corrected for diamagnetic contributions of the sample holder.^22^ Alternating current susceptibility measurements were taken of powdered samples to determine the in-phase and out-of-phase components of the magnetic susceptibility. The data were collected by increasing temperature from 2 K to 10 K, with no applied external dc field and a drive frequency of 2.5 Oe, with frequencies between 10 and 1000 Hz. In the samples where free movement of crystallites were prevented, silicone grease was employed for the embedding.

### Single-crystal X-ray crystallography

Diffraction data for these complexes were collected on a Bruker SMART CCD diffractometer (Mo Kα radiation and *λ* = 0.71073 Å) in *Φ* and *ω* scan modes. The structures were solved by direct methods followed by difference Fourier syntheses, and then refined by full-matrix least-squares techniques on *F*^2^ using SHELXL-97.^23^ All other non-hydrogen atoms were refined with anisotropic thermal parameters. Hydrogen atoms were placed in calculated positions and refined isotropically using a riding model. X-ray crystallographic data and refinement details for the complexes are summarized in Table S1.† Full details can be found in the CIF files provided in the ESI.†

#### Synthesis of [Dy_6_(L^1^)_4_(μ_3_-OH)_4_(MeOH)_4_]Cl_2_·2MeOH·2MeCN (1)

A mixture of DyCl_3_·6H_2_O (0.5 mmol, 0.189 g), H_3_L^1^ (0.25 mmol, 0.0895 g), and triethylamine (0.1 mmol) in 12 mL mixed solvent of MeOH–MeCN (v/v = 1 : 1) was stirred at room temperature for 30 min (pH = 7.6), and then the final mixture was sealed in a 25 mL Teflon-lined autoclave and heated at 80 °C for five days. After cooling to room temperature slowly, yellow block crystals of 1 were isolated (the yield based on DyCl_3_·6H_2_O: 42%). Elemental analysis calcd (%) for 1, C_86_H_110_Dy_6_N_10_O_30_Cl_2_: C, 56.30; H, 6.04; N, 7.63. Found: C, 56.51; H, 6.12; N, 7.73. IR (KBr disk, cm^−1^): 3408 (s), 3056 (w), 2967 (m), 1642 (m), 1558 (s), 1482 (s), 1421 (s), 1376 (m), 1231 (s), 1018 (m), 896 (w), 736 (w), 599 (w).

#### Synthesis of [Dy_6_(HL^2^)_2_(μ_3_-OH)_2_(μ_3_-OCH_3_)_2_(piv)_10_(MeOH)_2_] (2)

A mixture of Dy(NO_3_)_3_·6H_2_O (0.5 mmol, 0.228 g), H_3_L^2^ (0.5 mmol, 0.157 g), pivalic acid (3.0 mmol, 0.306 g) and triethylamine (0.2 mmol) in 16 mL mixed solvent of MeOH–MeCN (v/v = 1 : 1) was stirred at room temperature for 60 min (pH = 6.7), and then the final mixture was sealed in a 25 mL Teflon-lined autoclave and heated at 120 °C for six days. After cooling to room temperature slowly, yellow block crystals of 2 were isolated (the yield based on Dy(NO_3_)_3_·6H_2_O: 31%). Elemental analysis calcd (%) for 2, C_76_H_124_Dy_6_N_2_O_34_: C, 35.31; H, 4.84; N, 1.08. Found: C, 35.46; H, 4.97; N, 1.13. IR (KBr disk, cm^−1^): 3406 (s), 2940 (w), 1642 (m), 1630 (w), 1482 (w), 1467 (m), 1385 (s), 1295 (m), 1218 (m), 1158 (w), 1073 (m), 896 (w), 742 (w), 599 (w).

## Results and discussions

### Syntheses

Both complexes were synthesized under solvothermal conditions. Reactions of DyCl_3_·6H_2_O with H_3_L^1^ in the presence of Et_3_N in 10 : 5 : 2 molar ratio in a mixed solvent of CH_3_OH and CH_3_CN (1 : 1) in Teflon-lined autoclave at 80 °C for five days produced yellow crystals of 1 in 42% yield. Utilization of single solvent could not yield the compound. The Schiff base ligand in the complex has a −3 charge with all hydroxyl groups deprotonated. Reactions of Dy(NO_3_)_3_·6H_2_O, H_3_L^2^, pivalic acid, and Et_3_N in 5 : 5 : 30 : 2 molar ratio in similar solvent as 1 in Teflon-lined autoclave at 120 °C for six days produced yellow crystals of 2 in 31% yield. The ligand in complex 2 has a −2 charge with the phenolic hydroxyl group and one alcoholic hydroxyl group deprotonated. Reactions of H_3_L^1^ or H_3_L^2^ with other Dy(iii) sources, such as Dy(NO_3_)_3_·6H_2_O for H_3_L^1^ and DyCl_3_·6H_2_O for H_3_L^2^, have been tested, but no crystals of 1 and 2 were obtained.

### Crystal structures

The solid state structures of 1–2 were determined by single crystal X-ray crystallographic studies. Crystallographic data for complexes 1–2 are presented in Table S1,† and selected bond lengths and angles are given in Tables S3–S5† in the ESI.†

**Fig. 1:**
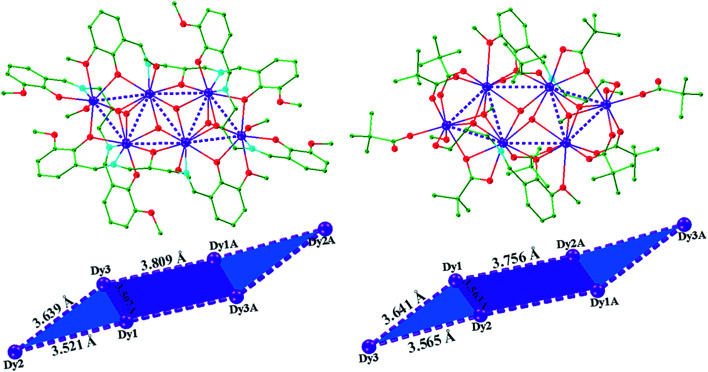
Crystal structure and chair-shaped of complex 1 (left) and 2 (right); hydrogen atoms and solvents are omitted for clarity. Color scheme: Dy, purple; O, red; N, light blue; C, green.

**Scheme 2:**
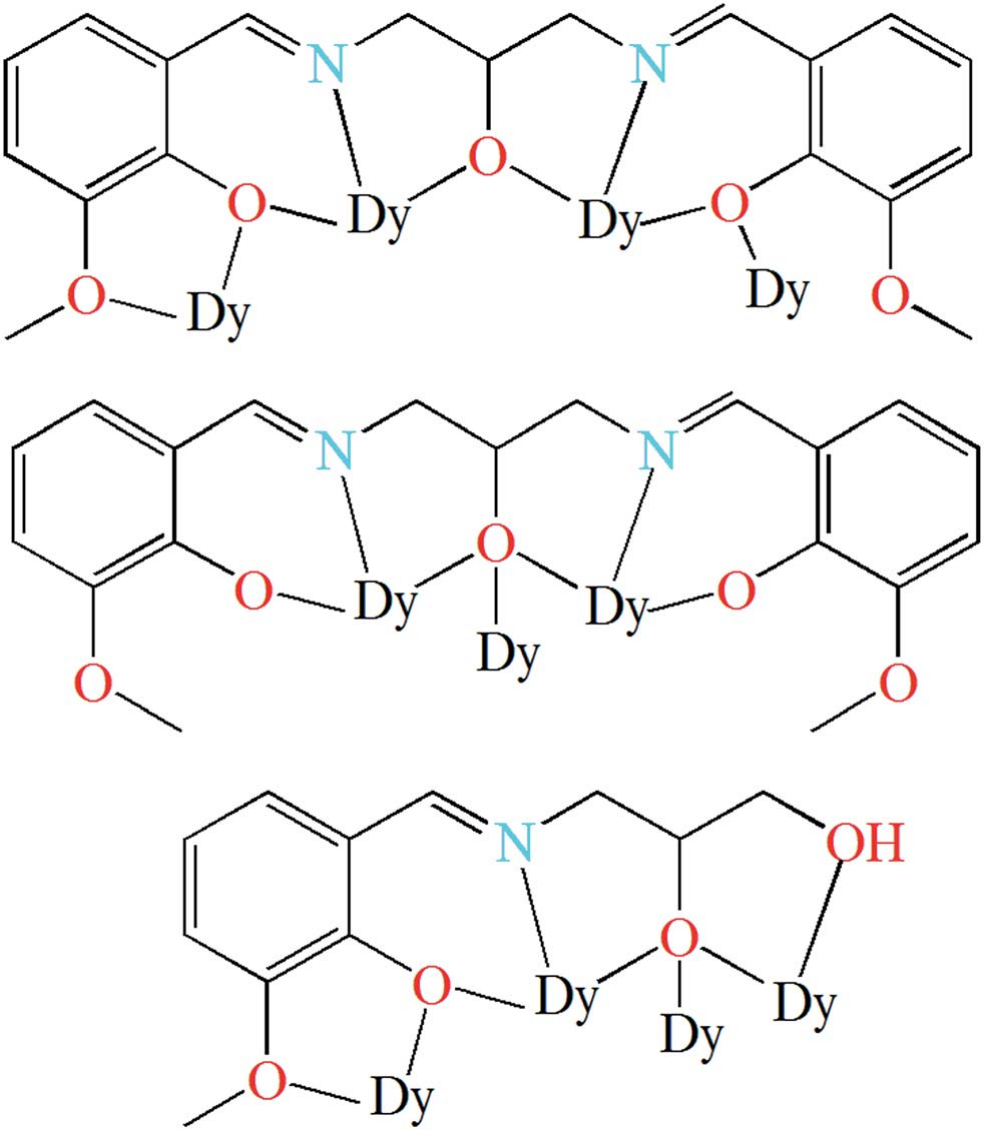
The coordination modes of (L^1^)^3−^ in 1 (top (μ_4_-L^1^-κ^9^O^1^,O^2^:O^2^,N^1^,O^3^:O^3^,N^2^,O^4^:O^4^) and middle (μ_3_-L^1^-κ^7^O^2^,N^1^,O^3^:O^3^:O^3^,N^2^,O^4^)) and (HL^2^)^2−^ in 2 (down (μ_4_-L^2^-κ^8^O^1^,O^2^:O^2^,N^1^,O^3^:O^3^:O^3^,O^4^)).

Single-crystal X-ray studies revealed that complex 1 crystallized in the triclinic space group *P*1̄, with the asymmetric unit containing the entire molecule as well as a chloride counterion and MeOH and MeCN solvent molecules. A view of the cationic complex 1 is shown in Fig. 1. The X-ray structure of 1 reveals a centrosymmetric core with two equivalent Dy_3_ moieties linked by two μ_3_-OH^−^ anions. Each μ_3_-OH^−^ anion links three Dy ions, and the Dy–Dy separations are similar at 3.521 Å, 3.507 Å and 3.639 Å for Dy1–Dy2, Dy1–Dy3 and Dy2–Dy3, respectively. Dy1 is coordinated with six oxygen atoms and two nitrogen atoms, which originating from two different L^1^ ligands, and two μ_3_-OH^−^ anions. The Dy2 center is ligated with seven oxygen atoms (Dy–O = 2.047(6)–2.235(5) Å) and one nitrogen atom (Dy–N = 2.235(5) Å) from three different Schiff-base ligands, one μ_3_-OH^−^ anion, and one MeOH molecule. Dy3 is coordinated with seven oxygen atoms and one nitrogen atom from two Schiff-base ligands, three μ_3_-OH^−^ anions, one MeOH molecule. All Dy ions are 8-coordinate and display distorted bicapped trigonal prism geometry. The hexanuclear Dy(iii) complex contain four Dy_3_ triangles. The Dy1, Dy3, Dy1A, and Dy3A atoms are in the same plane, forming a parallel quadrilateral, which containing two Dy_3_ triangles (Dy1, Dy3, Dy1A, and Dy1A, Dy3A, Dy1). Each triangle link by one μ_3_-OH^−^ anion. Dy2 and Dy2A are located at both ends of the parallel quadrilateral, Dy2A lying above the plane and Dy2 below. Therefore, two other Dy_3_ triangles (Dy1, Dy2, Dy3, and Dy1A, Dy2A, Dy3A) are formed. The latter two Dy_3_ triangles are linked *via* one μ_3_-bridging hydroxyl group of the ligand L^1^ and one μ_3_-OH^−^ (located on the front and back of the triangle). Obviously, the layout of the six Dy atoms forms a chair-shaped. Notably, such a hexanuclear Dy(iii) complex is quite different from other known examples.^24^ First, there exit a parallel quadrilateral containing two Dy_3_ triangles. Second, all Dy atoms are 8-coordinate with distorted bicapped trigonal prism geometry. Furthermore, the Schiff-base ligand has a −3 charge with two phenolic hydroxyl groups and one backbone hydroxyl group deprotonated in 1. And the ligands adopt two different coordination fashions (Scheme 2). Adjacent molecules are segregated by these ligands, the shortest intermolecular Dy–Dy separation is 8.478 Å, indicating that the molecules are quite well isolated. Hydrogen atoms were determined by analyzing the coordination environment of oxygen atoms and hydrogen bonds were found in 1. There is a triply hydrogen-bonded among the chloride ion (Cl1) and the solvent methanol molecule (O15), the coordinated methanol (O13), μ_3_-OH (O1) (Fig. S1†). Meanwhile, there is a strong hydrogen bonds interaction between the solvent methanol molecule (O15) and coordinated oxygen atoms (O2) with the O2–H2⋯O15 distances of 2.853 Å.

**Scheme 3:**
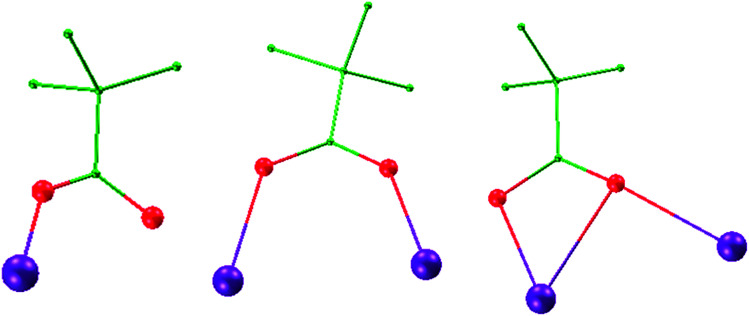
Three coordination modes of pivalate ligands in complex 2 (pivalate-κO, left; μ_2_-pivalate*-*κ^2^O^1^:O^2^, middle; μ_2_-pivalate*-*κ^3^O^1^O^2^:O^2^, right). Hydrogen atoms are omitted for clarity. Color scheme: Dy, purple; O, red; C, green.

A perspective view of 2 is depicted in Fig. 1. Complex 2 crystallises in the monoclinic space group *P*2_1_/*c*. The hexanuclear complex surrounded by two Schiff-base ligands and ten pivalate ligands. Only one coordination and bridging mode can be observed for the unique polydentate Schiff-base HL^2^ ligand (Scheme 2). Three different coordination modes of pivalate ligands coexist in the crystal structure (Scheme 3). Six of the ten pivalate anions acts as a bridging ligand with two oxygen atoms coordinating separately to two Dy ions. Two pivalate anions coordinate to two Dy ions respectively in μ_2_-pivalate*-*κ^3^O^1^O^2^:O^2^ fashion. The other two pivalate anions coordinate respectively to Dy ions in monodentate mode. Two methanol molecules occupy the remaining coordination sites of the Dy2 and Dy2A metal centres. The Dy1 is nine-coordinate with distorted capped square antiprism geometries (Fig. S2†), Dy2 and Dy3 are eight-coordinate, displaying triangular dodecahedron and square antiprism geometry, respectively. In the hexanuclear unit, Dy1, Dy2, Dy1A and Dy2A ions are present in the same plane, forming a parallel quadrilateral, whereas the Dy3 and Dy3A ions are arranged in a *trans* geometry with respect to each other and are displaced by 1.618 Å from the plane. Thus, the hexanuclear core adopts the chair conformation. This Dy_6_ complex also contain four Dy_3_ triangles. The two Dy_3_ triangles (Dy1, Dy2, Dy1A, and Dy1, Dy2A, Dy1A) are linked by one μ_3_-OH^−^ anion, respectively. Two other Dy_3_ triangles (Dy1, Dy2, Dy3, and Dy1A, Dy2A, Dy3A) are linked *via* one μ_3_-bridging hydroxyl group of the ligand L^2^ and one μ_3_-OCH_3_. The closest intercluster Dy–Dy distances is 9.49 Å, also indicating that the molecules are quite well isolated.

Up to now, many hexanuclear Dy^III^ complexes with different topologies have been reported (Table S2†). The arrangements of six Dy^III^ ions in the complex with ring (or wheel),^25^ hollow,^26^ hemi-cubane,^27^ trigonal prism,^28^ edge-to-edge,^17*c*,29^*etc.* have been described. The chair-shaped arrangement described here is newly-reported, and contributes an interesting example to the topology catalogues of the Dy_6_ complex.

Metal complexes of ligands H_3_L^1^ and H_3_L^2^ have been reported in literatures.^24,30–32^ For ligand H_3_L^1^, most of reported complexes, with 3d, 4f, 3d–4f, and others as metal centers, are finite multinuclear compounds.^24,30,31*a*–*f*^ Only two examples containing Cu ions are coordination polymers with one-dimensional structure which was built by the bridging of alcoholic hydroxyl group of the ligand.^31*g*,*h*^ The coordination modes of the ligand H_3_L^1^ in these reported complexes are summarized in Scheme S1.† For ligand H_3_L^2^, 3d and 4f metal complexes are reported, which are all finite multinuclear.^32^ The ligand in the complexes is found to show a variety of coordination modes that are listed in Scheme S2.† It is interesting to note that the coordination modes of the ligands H_3_L^1^ and H_3_L^2^ exhibited in this work (Scheme 1) have never been reported previously.

### Spectroscopic and thermal analyses of complexes 1–2

The infrared (IR) spectra of complexes 1–2 have been recorded between 4000 and 400 cm^−1^ with KBr pellets (Fig. S3–4†). In the low-wavenumber region, the spectra exhibit the characteristic Dy–O and Dy–N vibration bands. The characteristic strong absorptions at ∼1642 cm^−1^ were ascribed to the vibration of C=N bond, indicating the formation of the Schiff-base ligand.^33^ Furthermore, the resonances at 2967 and 2940 cm^−1^ are assigned to the *ν*(CH_2_) stretching vibrations, and the signals at 1482–1421 cm^−1^ and 1467–1385 cm^−1^ correspond to the *δ*(CH_2_) bending vibrations, respectively.^34^ The peaks appearing in the range of 1640–1390 cm^−1^ are assigned to the stretch of benzene ring from Schiff-base ligands. The *ν*(O–H) vibration at around 3400 cm^−1^ can also be observed in complexes 1–2, respectively. In summary, the results of the IR spectras are consistent with the single-crystal structural analyses.

Thermal analyses have been carried out to examine the thermal stability of the complexes 1–2. The crushed single-crystal samples were heated up to 1000 °C in N_2_ at a heating rate of 10 °C min^−1^. The TG curves of 1 (Fig. S5†) show that the first weight loss of 7.6% between 30 and 155 °C corresponds to the loss of solvent MeCN, MeOH and chloride counterion (calc. 7.8%). The weight gradually decrease 4.3% with temperature increasing from 155 and 203 °C corresponds to the loss of terminal MeOH molecules (calc. 4.5%). The residue is stable up to 213 °C, where after pyrolysis of Schiff-base ligand and then ends at 710 °C (weight loss: 45.3%; calc. 45.7%). The final residual weight of 42.5% (calc. 46.7%) corresponds to that of Dy_2_O_3_. The above thermal analysis results basically agree with the formula of 1. The thermal properties of the complex 2 was also investigated. The weight loss of 4.9% is observed at 200 °C which maybe corresponding to the coordinated MeOH molecules and part of pivalate ligands. When the temperature continues rising, the Schiff-base ligands and other pivalate ligands begin to decompose in the 200 to 600 °C temperature range. Finally, the remaining weight of 43.8% is in agreement with the proportional weight (calc. 43.3%) of Dy_2_O_3_.

### Magnetic properties

**Fig. 2:**
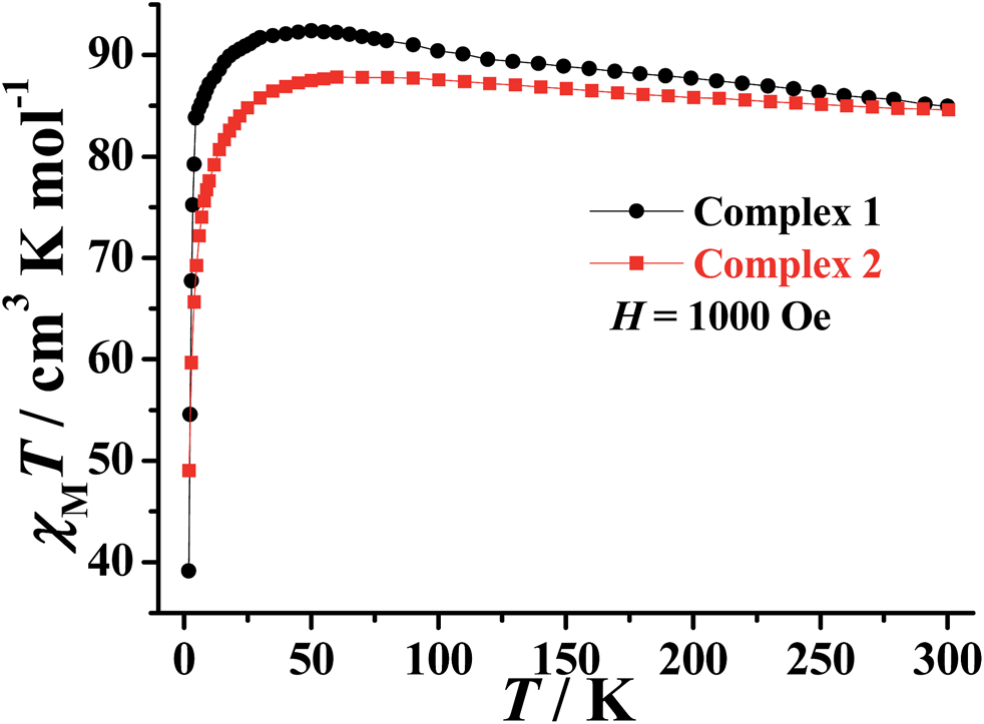
Plots of *χ*_M_*T vs. T* for 1–2 under applied dc magnetic field of 1000 Oe.

The direct-current (dc) magnetic susceptibility of 1–2 were measured in the range of 300–2 K with an applied magnetic field of 1000 Oe and can be seen plotted as *χ*_M_*T vs. T* in Fig. 2. The room temperature *χ*_M_*T* values of 84.95 and 84.57 cm^3^ K mol^−1^ for 1–2, respectively, which is in agreement with the expected value of 85.02 cm^3^ K mol^−1^ for six uncoupled Dy^III^ ions (Dy^III^: ^6^H_15/2_, *S* = 5/2, *L* = 5, *J* = 15/2, *g* = 4/3). The *χ*_M_*T* values of 1–2 gradually increase with decreasing temperature to reach a maximum of 92.39 cm^3^ K mol^−1^ at 50 K and 87.81 cm^3^ K mol^−1^ at 60 K, before decreasing to 39.12 cm^3^ K mol^−1^ and 49.01 cm^3^ K mol^−1^ at 2 K, respectively. The first increase of the *χ*_M_*T* values suggests the presence of a dominant intramolecular ferromagnetic interactions between the Dy^III^ ions. The low temperature decrease may be due to a combination of intermolecular antiferromagnetic interactions, large magnetic anisotropy and thermal population of the excited states of the Dy^III^ ions (Stark sublevels of the ^6^H_15/2_ state).^35^

**Fig. 3:**
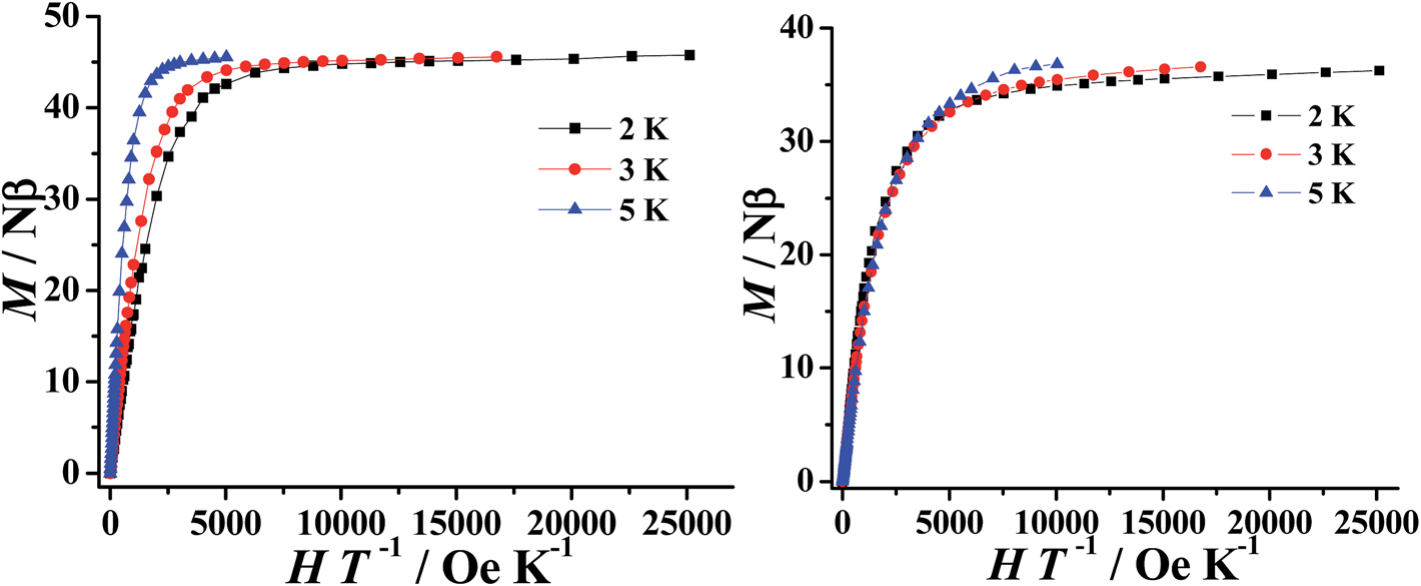
*M vs. H*/*T* plot at various temperatures for complexes 1 (left) and 2 (right).

The field dependence of the magnetization (*M*) for 1–2 were performed in the field (*H*) range of 0–5 T at 2–5 K. As expected for ferromagnetically coupled spins, the *M vs. H* data below 5 K reveal a rapid increase of the magnetization at low magnetic fields (Fig. S6†). At high fields, *M* increases rapidly to reach 45.76 *Nβ* and 36.38 *Nβ* at 2 K and under 5 T, respectively. These values are all lower than the corresponding theoretical saturated values. The non-superposition of the *M vs. H*/*T* data (Fig. 3) on a single master curve and the highfield non-saturation suggests the presence of significant magnetic anisotropy and/or low-lying excited states in complexes 1–2.^36,37^

**Fig. 4:**
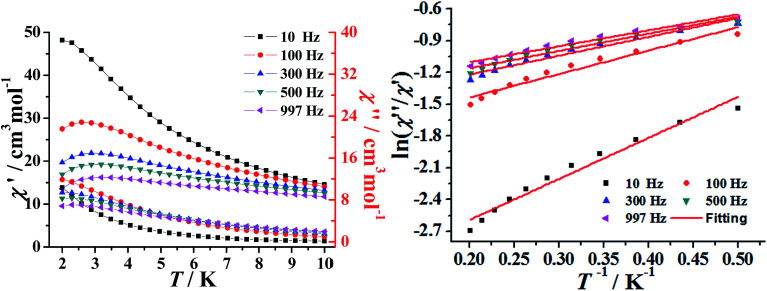
Temperature dependent ac susceptibility for 1 in the absence of a dc field (*H*_ac_ = 2.5 Oe) (left); plots of ln(*χ*′′/*χ*′) *vs.* 1/*T* for 1. The solid lines represent the fitting results over the temperature range of 2–5 K (right).

The temperature-dependent alternating-current (ac) susceptibility of complexes 1–2 were measured under zero-dc field and 2.5 Oe ac field (Fig. 4 and S7†). Both the in-phase (*χ*′) and out-of-phase (*χ*′′) signals of 1–2 show frequency dependence at low temperatures 2–10 K. The shape and the frequency dependence of the ac susceptibility signal indicate the slow magnetic relaxation behavior of 1–2. Although such ac signals were observed above 2 K, no obvious hysteresis was detected in the *M vs. H* data obtained using a traditional SQUID magnetometer (Fig. S8–9†).

The above magnetic determination indicates that complexes 1–2 display a clear signature of slow magnetic relaxation behavior. Unfortunately, the expected maximum due to the blocking could not be observed above 2 K, even by applying a dc magnetic field up to 1000 and 2000 Oe (Fig. S10†). Under the assumption that the SMM relaxation has just one characteristic time, we could obtain the energy barriers and *τ*_0_ values by fitting the ac susceptibility data from adopt the Debye model.^37^ This gave *τ*_0_ = 9.03 × 10^−5^ s and *U*_eff_ = 1.03 K for 1 and *τ*_0_ = 2.36 × 10^−5^ s and *U*_eff_ = 4.21 K for 2 (Fig. 4 and S7†). The Cole–Cole diagram can be used to study the distribution of the relaxation process, which is frequently characterized and discussed for SMMs. The data of 1–2 plotted as Cole–Cole diagrams are shown in Fig. S11.† The shape of the Cole–Cole plot of 1–2 are relatively symmetrical and can be fitted to the generalized Debye model with a parameter *α* ranging from 0.11 to 0.15 for 1, 0.07–0.13 for 2, respectively, the relatively small *α* value indicates that a single relaxation time is mainly involved in the present relaxation process independently of the temperature.

## Conclusions

In summary, we have synthesized two hexanuclear Dy^III^ complexes based on two polyhydroxy Schiff-base ligands. It is interested that the complexes can be regarded as the result of the construction with Dy_3_ triangular motifs as building blocks, and the six Dy^III^ ions in the complex are arranged in a newly-reported chair-shaped geometry. The measurements of the dc susceptibility and of the field dependence of magnetization indicated the existence of the intramolecular ferromagnetic interactions. The frequency-dependent ac susceptibility signals revealed by the ac susceptibility investigations evidenced the SMM behaviors under zero dc field for two compounds. The lanthanide SMMs exhibiting intramolecular ferromagnetic interactions are rarely reported, and the synthesis of such a lanthanide SMM is believed to be of great challenges. The results of this work might imply an effective approach for the rational design and construction of lanthanide SMMs with novel magnetic behaviors by means of the lanthanide building blocks.

## Conflicts of interest

There are no conflicts to declare.

## Supplementary Material

RA-008-C7RA11378A-s001

RA-008-C7RA11378A-s002
