# Supplementary material for: Structure and magnetism of two chair-shaped hexanuclear dysprosium(iii) complexes exhibiting slow magnetic relaxation
Source: RSC Adv. 2018 Jan 3;8(2):767–74. doi: 10.1039/c7ra11378a (PMC9076819; doi:10.1039/c7ra11378a)
Supplement: RA-008-C7RA11378A-s001 [file RA-008-C7RA11378A-s001.pdf]

Electronic Supplementary Material (ESI) for RSC Advances.
This journal is © The Royal Society of Chemistry 2017

Electronic Supplementary Information (ESI)

Structure and Magnetism of Two Chair-Shaped Hexanuclear Dysprosium(III) Complexes Exhibiting Slow Magnetic Relaxation

Zi-Yuan Liu [a], Hua-Hong Zou [a,*], Rong Wang [a], Man-Sheng Chen [a], Fu-Pei Liang [a,b,*]

[a]*State Key Laboratory for Chemistry and Molecular Engineering of Medicinal Resources, School of Chemistry & Pharmacy of Guangxi Normal University, Guilin 541004, P. R. China, E-mail: gxnuchem@foxmail.com*

[b]*Guangxi Key Laboratory of Electrochemical and Magnetochemical Functional Materials, College of Chemistry and Bioengineering, Guilin University of Technology, Guilin 541004, P. R. China, E-mail: fliangoffice@yahoo.com*

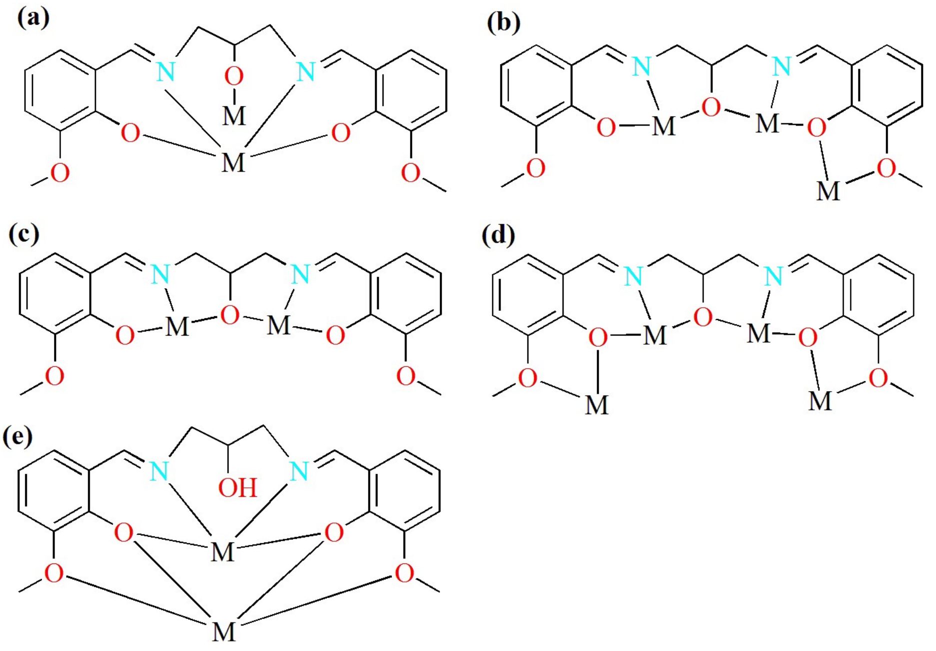

Scheme S1. The crystallographically up-to-date established coordination modes of the H_3L^1 ligand: (a) μ_2-L^1-$\kappa^5O^2,N^1:N^2,O^4:O^3$; (b) μ_3-L^1-$\kappa^8O^2,N^1,O^3:O^3,N^2,O^4:O^4,O^5$; (c) μ_2-L^1-$\kappa^6O^2,N^1,O^3:O^3,N^2,O^4$; (d) μ_4-L^1-$\kappa^{10}O^1,O^2:O^2,N^1,O^3:O^3,N^2,O^4:O^4,O^5$; (e) μ_2-L^1-$\kappa^8O^1,O^2,O^4,O^5:O^2,O^4,N^1,N^2$.

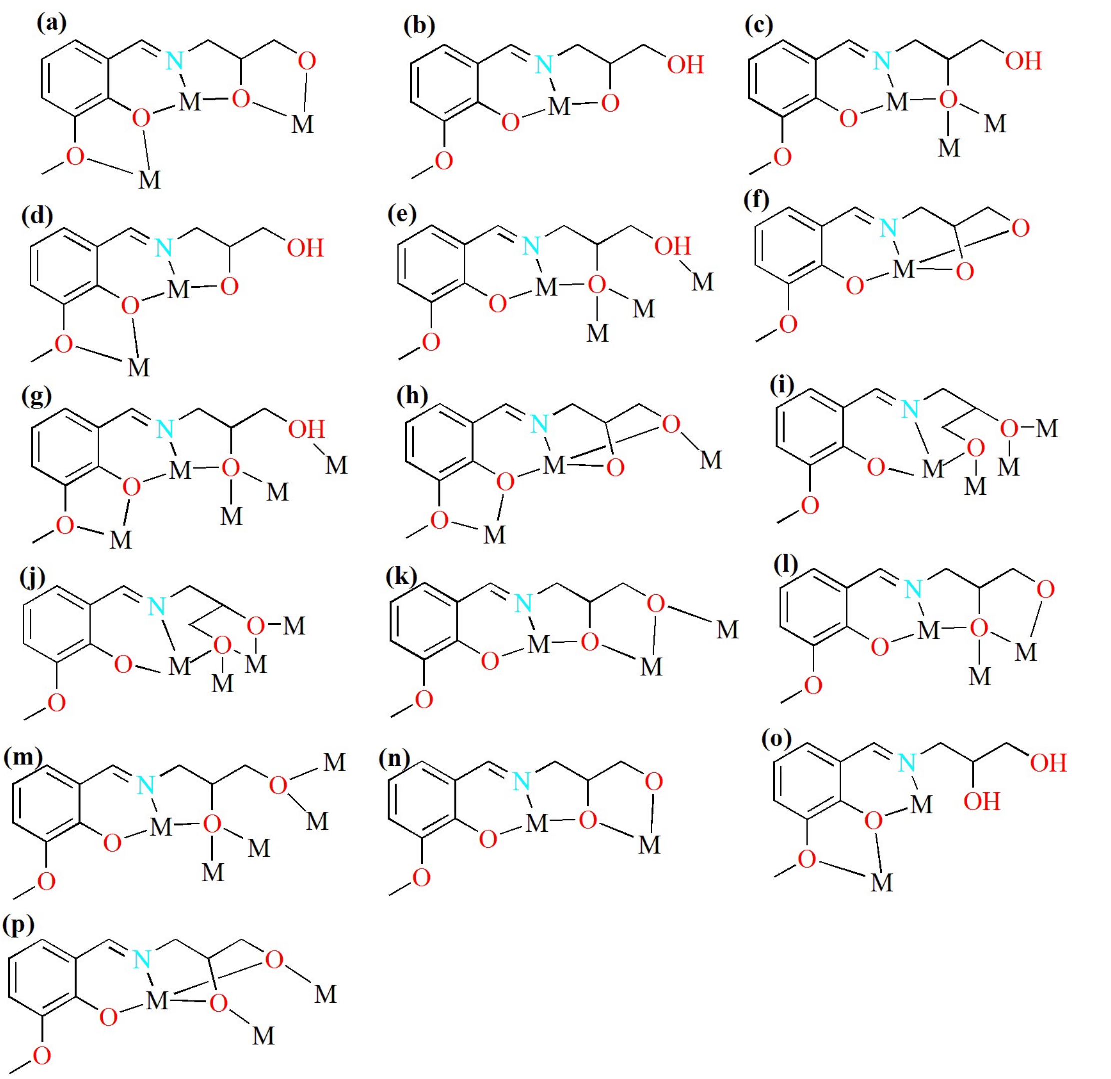

Scheme S2. The crystallographically up-to-date established coordination modes of the H_3L^2 ligands: (a) μ_3-L^2-$\kappa^7O^1,O^2:O^2,N^1,O^3:O^3,O^4$; (b) L^2-κ^3O^2,N^1,O^3; (c) μ_3-L^2-$\kappa^5O^2,N^1,O^3:O^3:O^3$; (d) μ_2-L^2-$\kappa^5O^1,O^2:O^2,N^1,O^3$; (e) μ_4-L^2-$\kappa^6O^2,N^1,O^3:O^3:O^3:O^4$; (f) L^2-κ^4O^2,N^1,O^3,O^4; (g) μ_5-L^2-$\kappa^8O^1,O^2:O^2,N^1,O^3:O^3:O^3:O^4$; (h) μ_3-L^2-$\kappa^7O^1,O^2:O^2,N^1,O^3,O^4:O^4$; (i) μ_4-L^2-$\kappa^6O^2,N^1,O^4:O^3:O^3:O^4$; (j) μ_4-L^2-$\kappa^7O^2,N^1,O^4:O^3:O^3,O^4:O^4$; (k) μ_3-L^2-$\kappa^6O^2,N^1,O^3:O^3,O^4:O^4$; (l) μ_3-L^2-$\kappa^6O^2,N^1,O^3:O^3:O^3,O^4$; (m) μ_5-L^2-$\kappa^7O^2,N^1,O^3:O^3:O^3:O^4:O^4$; (n) μ_2-L^2-$\kappa^5O^2,N^1,O^3:O^3,O^4$; (o) μ_2-L^2-$\kappa^4O^1,O^2:O^2,N^1$; (p) μ_3-L^2-$\kappa^6O^2,N^1,O^3,O^4:O^3:O^4$.

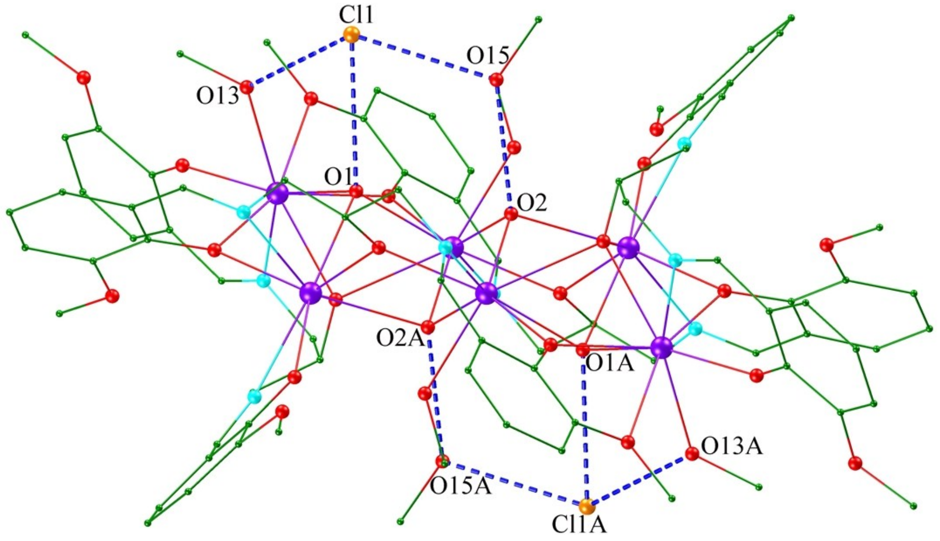

Figure S1. The hydrogen-bonded interaction of complex **1**, Color scheme: Dy, purple; O, red; N, light blue; C, green; Cl, light yellow; Hydrogen atoms and part of solvents are omitted for clarity.

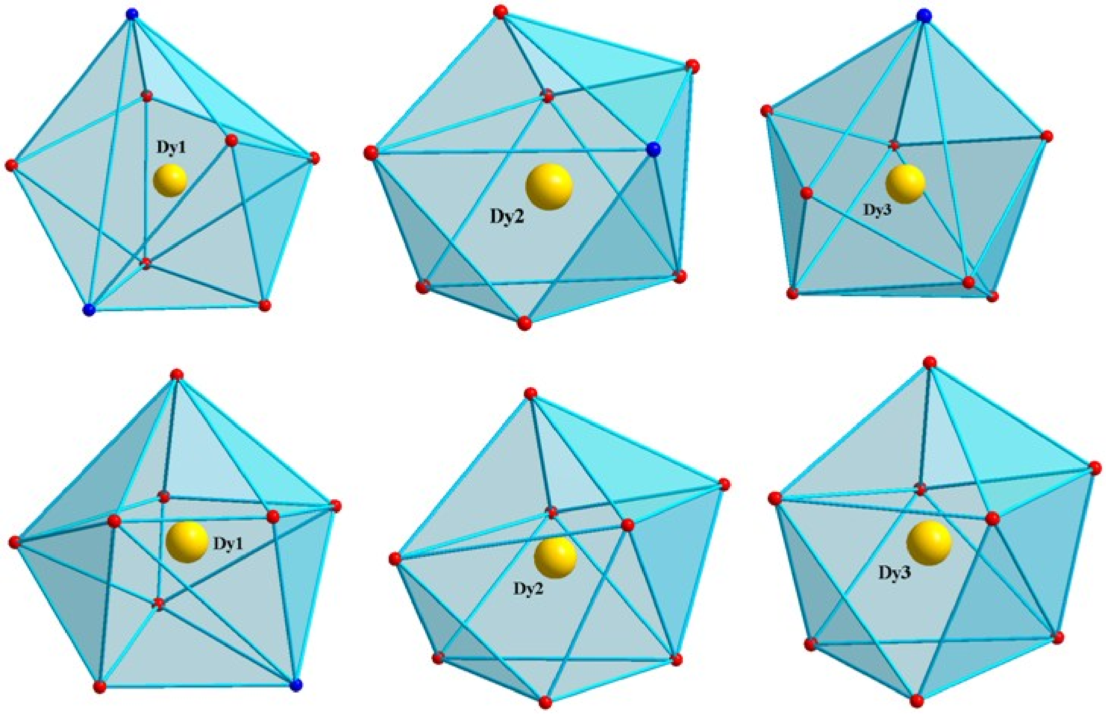

Figure S2. The coordination polyhedra of the Dy^{III} ions in complexes **1** (top) and **2** (down).

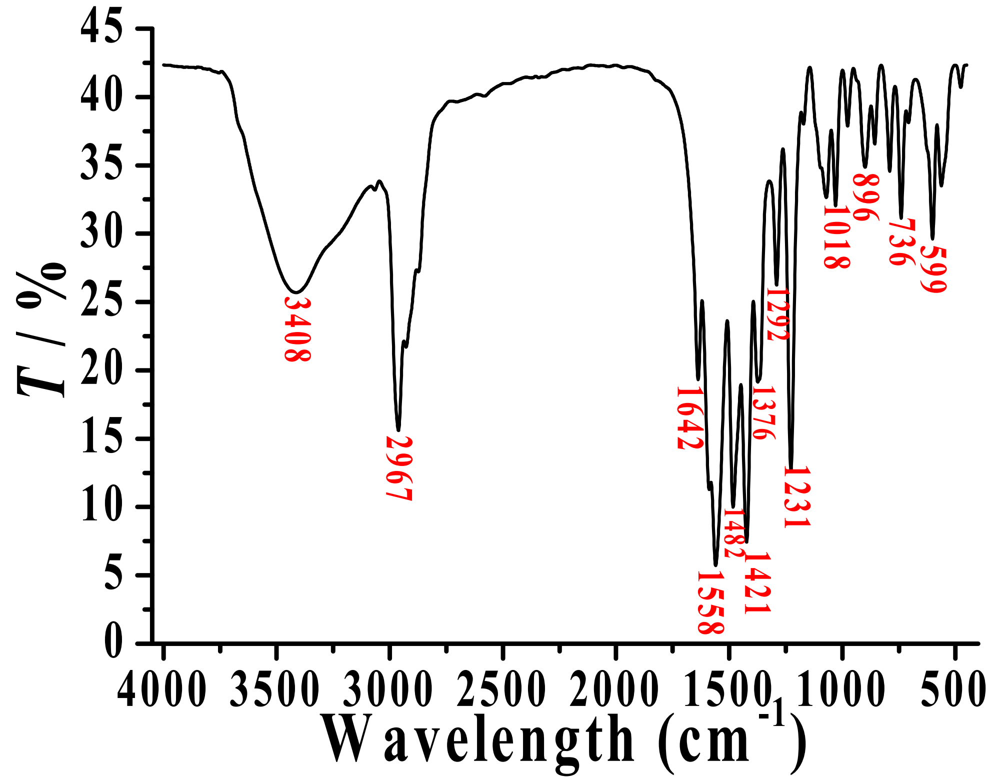

Figure S3. FT-IR spectrum of complex **1**.

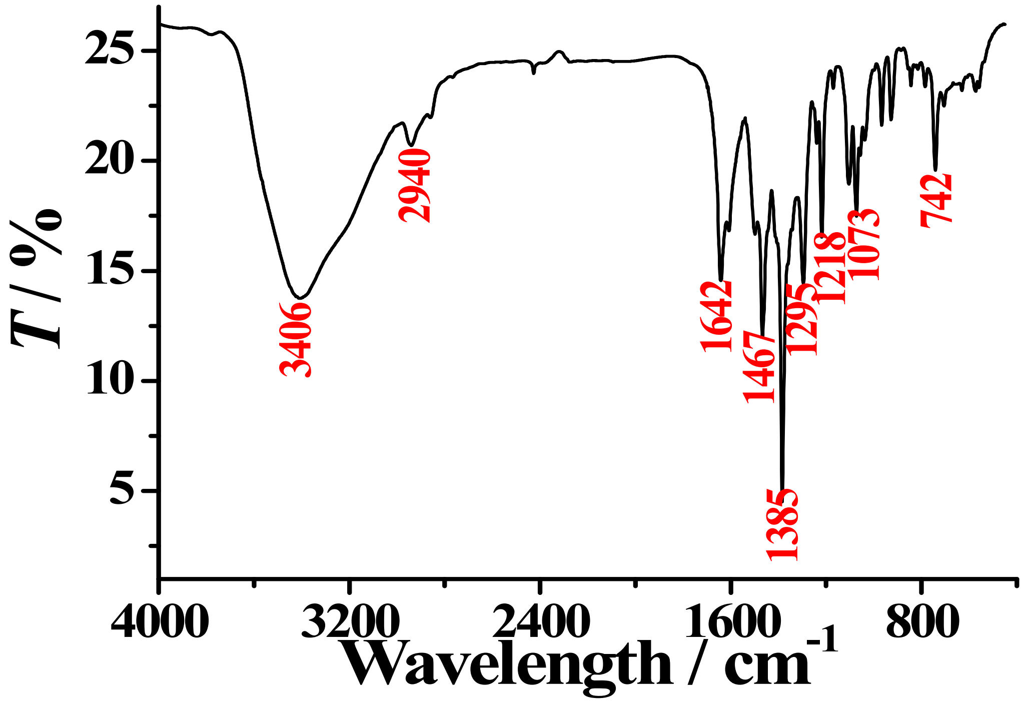

Figure S4. FT-IR spectrum of complex **2**.

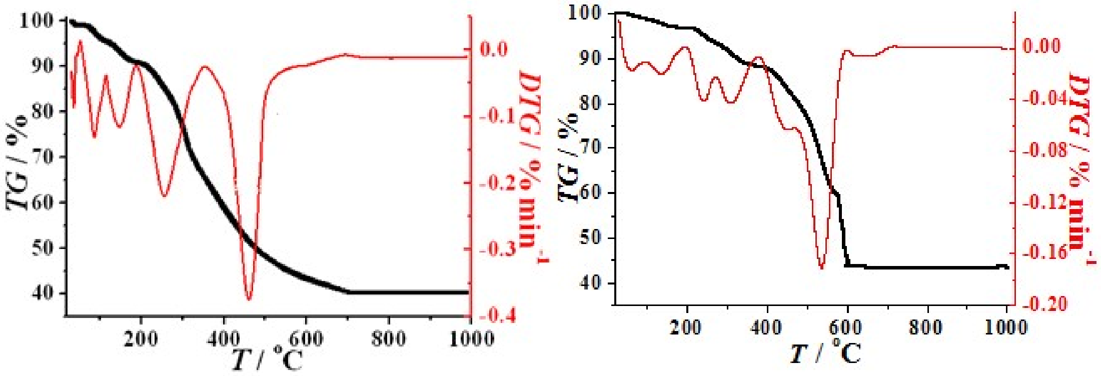

Figure S5. TG-DTG curve of complexes **1** (left) and **2** (right).

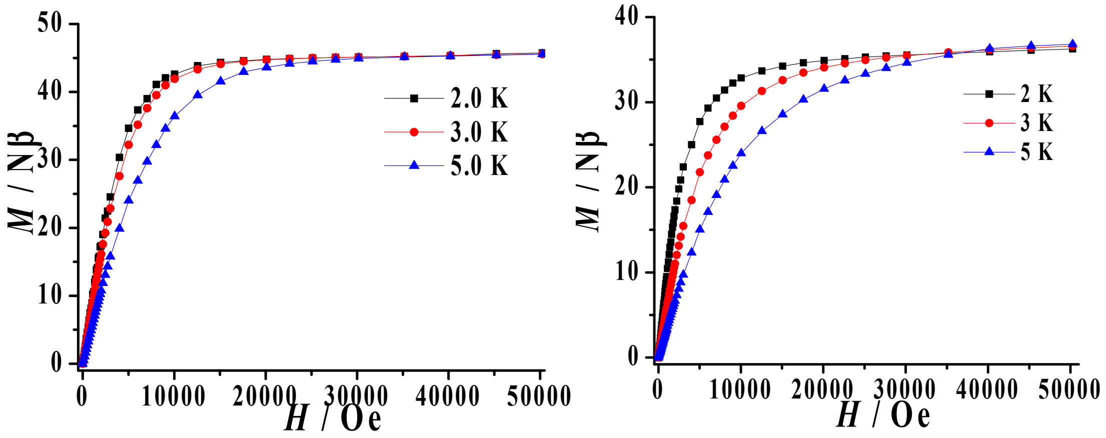

Figure S6. M *vs*. H plot at various temperatures for complexes **1** (left) and **2** (right).

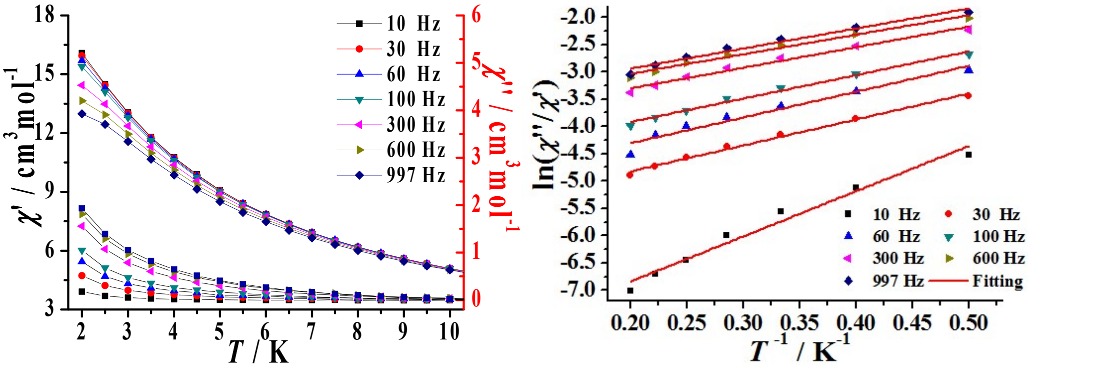

Figure S7. Temperature dependent ac susceptibility for **2** in the absence of dc field (H_{ac} = 2.5 Oe) (left); Plots of ln(χ''/χ') *vs*. $1/T$ for **2**. The solid lines represent the fitting results over the temperature range of 2–5 K (right).

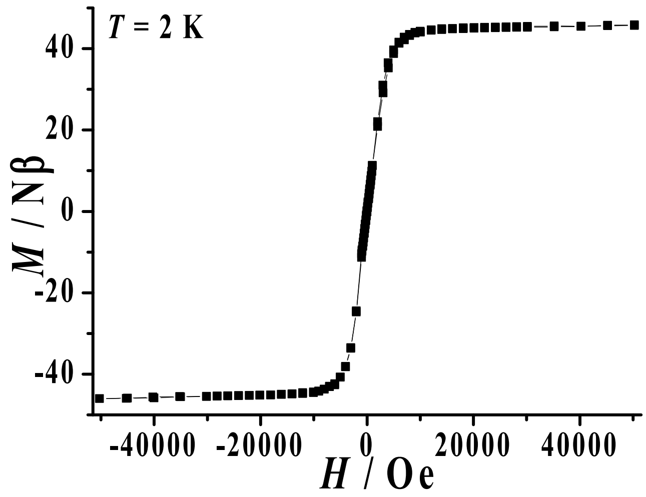

Figure S8. Magnetic hysteresis loop of **1** recorded at 2 K.

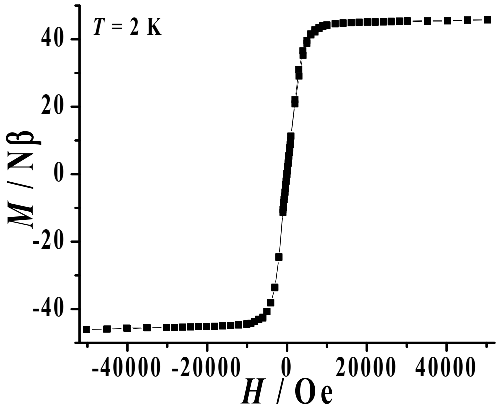

Figure S9. Temperature dependent out-of-phase ac susceptibility for **2** in the absence of a dc field (H_{ac} = 2.5 Oe). Magnetic hysteresis loop of **2** recorded at 2 K.

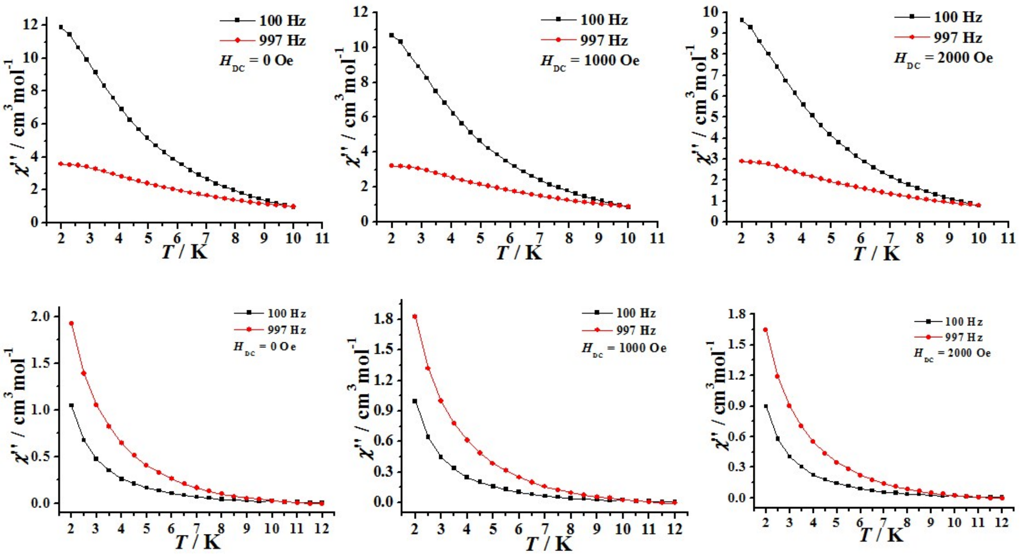

Figure S10. Temperature dependence of the out-of-phase susceptibility at 100 and 997 Hz and applied fields of 0 (left), 1000 (middle), and 2000 Oe (right) for the complexes **1** (up) and **2** (down).

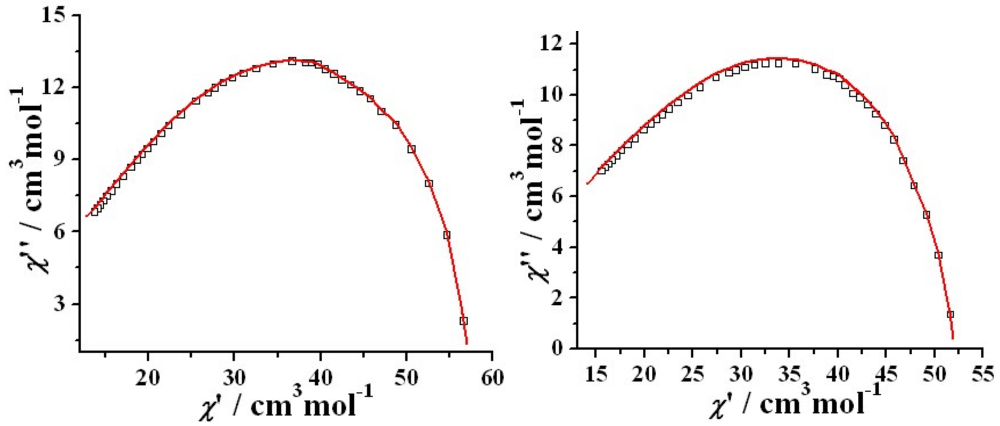

Figure S11. Cole-Cole diagram of **1-2** at 2 K with zero dc field; the solid lines are the best fits to the experimental data, obtained with the generalized Debye model.

Table S1. Details of the Data Collection and Refinement Parameters for Complexes **1−2**.

	Complex **1**	Complex **2**
Chemical formula	$C_{86}H_{110}Dy_6N_{10}O_{30}Cl_2$	$C_{76}H_{124}Dy_6N_2O_{34}$
Formula weight	2809.75	2584.78
Crystal system	Triclinic	Monoclinic
Space group	*P*-1 (No. 2)	$P2_1/c$ (No. 14)
a / Å	12.4846(3)	13.4065(3)
b / Å	14.5722(2)	21.7010(4)
c / Å	15.5223(2)	17.9936(4)
α / °	79.1570(10)	90
β / °	67.587(2)	99.840(2)
γ / °	69.131(2)	90
V / Å^3	2434.88(9)	5157.95(19)
Z	1	2
D_{calcd} / g cm^{-3}	1.916	1.664
μ / mm^{-1}	4.681	4.362
F(000)	1366	2524
T / K	153	153
Reflections total	16896	24217
Theta Min-Max [Deg]	2.8, 26.0	3.0, 25.1
Min-Max. Resd. Dens. [e Å^{-3}]	-1.39, 2.19	-1.13, 1.78
Reflections unique	9574	9168
R_{int}	0.026	0.033
GOF on F^2	1.04	1.18
R_1 [I>2σ(I)]	0.0306	0.0522
wR_2 (all reflections)	0.0729	0.1257
CCDC	1042130	1042131

Table S2. Different arrangement of the six Dy ions in Dy_6 complexes and related information.

**Type**	**Structure**	$\Delta E/k_B$ **[K]**	τ_0 **[s]**	**Ref**
Co-parallel, not coplanar	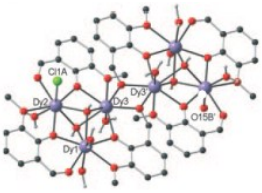	200	15×10^{-9}	[1]
Co-parallel, not coplanar	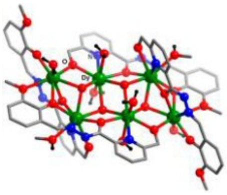	46.2	2.85×10^{-7}	[2]
Planar	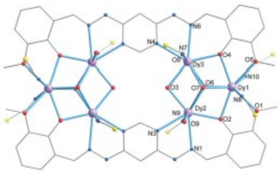	116<br>181	1.11×10^{-11}<br>8.1×10^{-9}	[3]
Planar	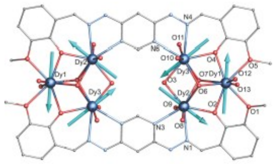	27<br>98	1.56×10^{-6}<br>5.25×10^{-7}	[4]
Edge-to-edge arrangement	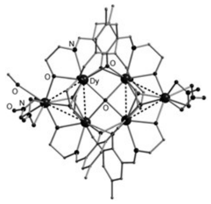	33.9<br>40.7	5.8×10^{-8}<br>1.2×10^{-7}	[5]
Edge-to-edge arrangement	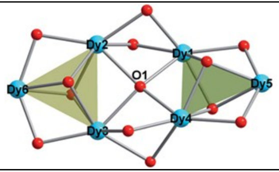	no	no	[6]
Hollow	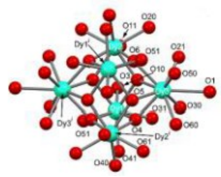	no	no	[7]
Hemi-cubanes	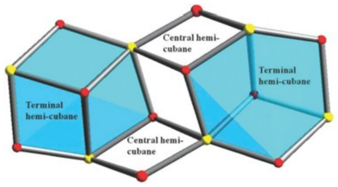	9.7 (H_{dc} = 1800 Oe)	6.4×10^{-6}	[8]

Ring	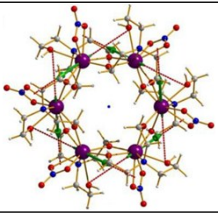	18.48	9.47×10^{-6}	[9]
Ring	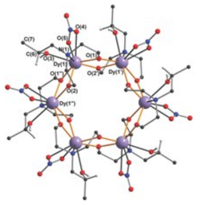	no	no	[10]
Ring	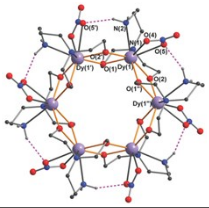	3<br><br>36<br>(H_{dc} = 2000 Oe)	2.7×10^{-5}<br><br><br>5.2×10^{-8}	[11]
Trigonal prism	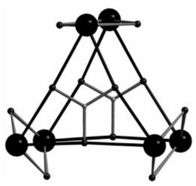	56	6.6×10^{-6}	[12]
Trigonal prism	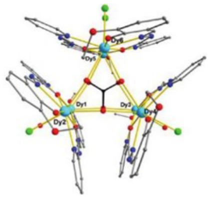	76	1.2×10^{-6}	[13]
Wheel	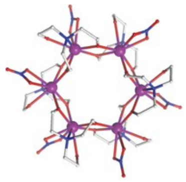	no	no	[14]
Wheel	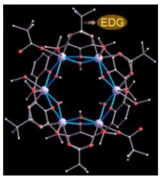	12.2	5.0×10^{-6}	[15]
Wheel	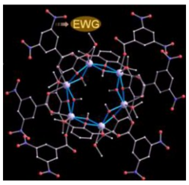	11.5	5.1×10^{-6}	[15]
Co-parallel	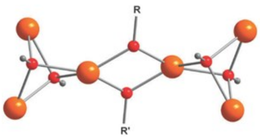	9.6	2×10^{-6}	[16]

Co-parallel	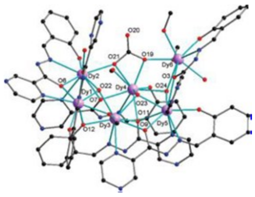	5.6<br>37.9	4.2×10^{-5}<br>3.8×10^{-6}	[17]
Vertex- and Edge-Sharing	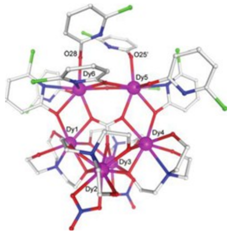	3.8	7.89×10^{-6}	[18]
Vertex- and Edge-Sharing	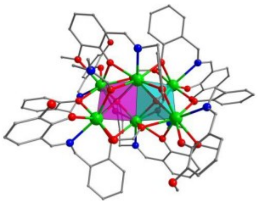	85	3.9×10^{-7}	[19]
Vertex- and Edge-Sharing	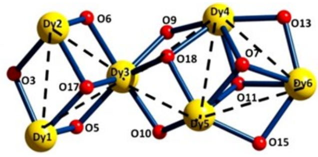	3.0	8.3×10^{-6}	[20]
Bowl	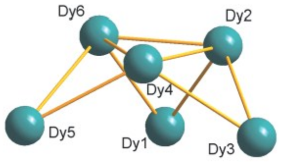	no	no	[21]
Butterfly	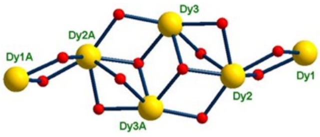	1.33<br>2.89	8.55×10^{-5}<br>1.73×10^{-5}	[22]
Sandwich-like	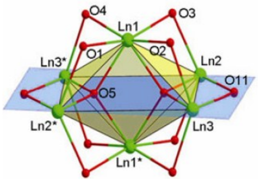	7.6	1.1×10^{-6}	[23]

References:

1 I. J. Hewitt, J. Tang, N. T. Madhu, C. E. Anson, Y. Lan, J. Luzon, M. Etienne, R. Sessoli and A. K. Powell, *Angew. Chem. Int. Ed.*, 2010, **49**, 6352.

2 S. Das, S. Hossain, A. Dey, S. Biswas, J.-P. Sutter and V. Chandrasekhar. *Inorg. Chem.*, 2014, **53**, 5020.

3 X. L. Li, H. Li, D. M. Chen, C. Wang, J. Wu, Ji. Tang, W. Shi and P. Cheng, *Dalton Trans.*, 2015, **44**, 20316.

4 X. L. Li, J. Wu, J. Tang, B. Le Guennic, W. Shi and P. Cheng, *Chem. Commun.*, 2016, **52**, 9570.

5 S. Y. Lin, W. Wernsdorfer, L. Ungur, A. K. Powell, Y. N. Guo, J. Tang, L. Zhao, L. F. Chibotaru and H. J. Zhang. *Angew. Chem. Int. Ed.*, 2012, **51**, 12767.
6 S. Y. Lin, J. F. Wu and Z. K. Xu, *RSC Adv.*, 2017, **7**, 47520.
7 T. T. da Cunha, F. Pointillart, B. Le Guennic, C. L. M. Pereira, S. Golhen, O. Cador and L. Ouahab. *Inorg. Chem.*, 2013, **52**, 9711.
8 A. Adhikary, J. A. Sheikh, S. Biswas and S. Konar. *Dalton Trans.*, 2014, **43**, 9334.
9 L. Ungur, S. K. Langley, T. N. Hooper, B. Moubaraki, E. K. Brechin, K. S. Murray and L. F. Chibotaru, *J. Am. Chem. Soc.*, 2012, **134**, 18554.
10 A. Baniodeh, N. Magnani, S. Bräse, C. E. Anson and A. K. Powell, *Dalton Trans.*, 2015, **44**, 6343.
11 H. Q. Tian, M. Wang, L. Zhao, Y. N. Guo, Y. Guo, J. Tang and Z. L. Liu, *Chem. Eur. J.*, 2012, **18**, 442 .
12 Y. N. Guo, X. H. Chen, S. F. Xue, and J. Tang, *Inorg. Chem.*, 2012, **51**, 4035.
13 S. K. Langley, B. Moubaraki, C. M. Forsyth, I. A. Gass and K. S. Murray. *Dalton Trans.*, 2010, **39**, 1705.
14 B. Joarder, S. Mukherjee, S. F. Xue, J. Tang, and S. K. Ghosh, *Inorg. Chem.*, 2014, **53**, 7554.
15 B. Hussain, D. Savard, T. J. Burchell, W. Wernsdorfer and M. Murugesu, *Chem. Commun.*, 2009, 1100.
16 H. Q. Tian, Y. N. Guo, L. Zhao, J. Tang,and Z. L. Liu, *Inorg. Chem.*, 2011, **50**, 8688.
17 S. K. Langley, B. Moubaraki, and K. S. Murray. *Inorg. Chem.*, 2012, **51**, 3947.
18 S. Mukherjee, A. K. Chaudhari, S. F. Xue, J. Tang and S. K. Ghosh, *Inorg. Chem. Commun.*, 2013, **35**, 144.
19 H. F. Zhang, R. Liu, J. Zhang, Y. Li, W. Liu and Y. Dong, *Chem. Asian J.*, 2017, **12**, 507.
20 L. J. Wu, H. Yang, S. Y. Zeng, D. C. Li and J. M. Dou, *Polyhedron.*, 2017, **129**, 77.
21 J. P. Costes and C. Duhayon, *Eur. J. Inorg. Chem.*, 2014, 4745.
22 J. P. Tong, F. Shao, M. G. Chen, Y. N. Tong, J. J. Zhuan, X. J. Xu, J. Tao and L. S. Zheng, *Inorg. Chem. Commun.*, 2016, **74**, 93.
23 Y. F. Bi, G. C. Xu, W. P. Liao, S. C. Du, R. P. Deng and B. W. Wang, *Sci. China. Chem.*, 2012, **55**, 967.

Table S3. Bond lengths and bond angles parameters for complex **1**.

Bond Length (Å)					
Dy1-O1	2.403(3)	Dy2-O1	2.362(4)	Dy3-O1	2.350(3)
Dy1-O3	2.275(4)	Dy2-O5	2.402(3)	Dy3-O2	2.378(4)
Dy1-O5	2.370(4)	Dy2-O6	2.187(3)	Dy3-O5	2.483(3)
Dy1-N1	2.524(5)	Dy2-O10	2.319(3)	Dy3-O10	2.364(3)
Dy1-O2a	2.366(3)	Dy2-O11	2.623(4)	Dy3-O12	2.257(3)
Dy1-O8a	2.356(3)	Dy2-O13	2.385(3)	Dy3-O14	2.368(4)
Dy1-O12a	2.304(4)	Dy2-N2	2.465(4)	Dy3-N3	2.532(5)
Dy1-N4a	2.511(6)	Dy2-O8a	2.345(3)	Dy3-O2a	2.384(3)
Bond Angle (°)					
O1-Dy1-O3	159.73(14)	O1-Dy2-O5	62.28(11)	O1-Dy3-O10	72.02(12)
O1-Dy1-O5	62.17(12)	O1-Dy2-O6	155.25(13)	O1-Dy3-O12	153.83(14)
O1-Dy1-N1	127.48(15)	O1-Dy2-O10	72.60(13)	O1-Dy3-O14	97.87(11)

O1-Dy1-O2a	80.25(10)	O1-Dy2-O11	110.24(12)	O1-Dy3-N3	139.74(14)
O1-Dy1-O8a	76.91(10)	O1-Dy2-O13	78.24(12)	O1-Dy3-O2a	80.97(11)
O1-Dy1-O12a	78.77(12)	O1-Dy2-N2	129.43(12)	O2-Dy3-O5	130.09(12)
O1-Dy1-N4a	86.81(15)	O1-Dy2-O8a	77.91(11)	O2-Dy3-O10	138.99(10)
O3-Dy1-O5	131.69(13)	O5-Dy2-O6	135.69(11)	O2-Dy3-O12	69.34(12)
O3-Dy1-N1	71.40(16)	O5-Dy2-O10	68.74(10)	O2-Dy3-O14	77.48(12)
O2a-Dy1-O3	90.33(13)	O5-Dy2-O11	129.36(11)	O2-Dy3-N3	133.26(13)
O3-Dy1-O8a	119.57(13)	O5-Dy2-O13	137.63(13)	O2-Dy3-O2a	69.67(11)
O3-Dy1-O12a	81.10(13)	O5-Dy2-N2	68.82(13)	O5-Dy3-O10	66.68(10)
O3-Dy1-N4a	87.85(17)	O5-Dy2-O8a	71.11(11)	O5-Dy3-O12	132.90(10)
O5-Dy1-N1	67.58(14)	O6-Dy2-O10	126.23(13)	O5-Dy3-O14	138.35(11)
O2a-Dy1-O5	71.81(12)	O6-Dy2-O11	72.93(12)	O5-Dy3-N3	92.02(13)
O5-Dy1-O8a	71.50(12)	O6-Dy2-O13	78.74(13)	O2a-Dy3-O5	69.55(11)
O5-Dy1-O12a	128.02(11)	O6-Dy2-N2	73.97(13)	O10-Dy3-O12	131.59(13)
O5-Dy1-N4a	135.73(15)	O6-Dy2-O8a	91.89(11)	O10-Dy3-O14	72.76(12)
O2a-Dy1-N1	98.28(13)	O10-Dy2-O11	61.89(11)	O10-Dy3-N3	69.71(13)
O8a-Dy1-N1	73.74(12)	O10-Dy2-O13	115.03(13)	O2a-Dy3-O10	135.59(10)
O12a-Dy1-N1	149.69(13)	O10-Dy2-N2	78.76(13)	O12-Dy3-O14	82.18(11)
N1-Dy1-N4a	121.94(17)	O8a-Dy2-O10	137.85(10)	O12-Dy3-N3	66.38(14)
O2a-Dy1-O8a	142.68(13)	O11-Dy2-O13	76.91(13)	O2a-Dy3-O12	85.02(11)
O2a-Dy1-O12a	68.77(12)	O11-Dy2-N2	90.64(13)	O14-Dy3-N3	82.43(14)
O2a-Dy1-N4a	136.52(16)	O8a-Dy2-O11	159.53(10)	O2a-Dy3-O14	147.11(13)
O8a-Dy1-O12a	133.14(11)	O13-Dy2-N2	152.33(13)	O2a-Dy3-N3	119.47(13)
O8a-Dy1-N4a	71.17(16)	O8a-Dy2-O13	86.87(13)	Dy2-O10-Dy3	101.97(14)
O12a-Dy1-N4a	68.03(15)	O8a-Dy2-N2	98.40(13)	Dy2-O5-Dy3	96.28(10)
Dy1a-O8-Dy2a	97.00(11)	Dy1a-O12-Dy3	113.28(15)	Dy2-O1-Dy3	101.11(15)
Dy1a-O2-Dy3	106.86(15)	Dy1-O5-Dy2	95.11(12)	Dy3-O2-Dy3a	110.33(12)
Dy1a-O2-Dy3a	95.19(10)	Dy1-O5-Dy3	92.52(13)		

Table S4. Hydrogen bond lengths (Å) and angles (°) for complex **1**.

D–H···A	*d*(D–H)	*d*(H···A)	*d*(D···A)	∠(DHA)	Symmetry codes
O1–H1···Cl1	0.8500	2.4600	3.281(4)	163.00	-1+x, y, z
O2–H2···O15	0.8500	2.0200	2.853(6)	165.00	
O13–H13A···Cl1	0.8500	2.2400	3.033(5)	156.00	-1+x, y, z
O14–H14B···O3	0.8500	1.9800	2.674(6)	138.00	-x, -y, 1-z
O14–H14B···O4	0.8500	2.4800	3.219(6)	146.00	-x, -y, 1-z
O15–H15D···Cl1	0.8500	2.2200	3.068(5)	175.00	-1+x, y, z

Table S5. Bond lengths and bond angles parameters for complex **2**.

Bond Length (Å)					
Dy1-O1	2.414(13)	Dy2-O8	2.344(11)	Dy3-O2	2.366(11)
Dy1-O6	2.589(12)	Dy2-O9	2.319(11)	Dy3-O3	2.274(12)
Dy1-O12	2.336(11)	Dy2-O13	2.415(8)	Dy3-O4	2.286(11)

Dy1-O13	2.407(9)	Dy2-O16	2.364(9)	Dy3-O6	2.358(11)
Dy1-O14	2.301(8)	Dy2-O5a	2.677(11)	Dy3-O7	2.476(13)
Dy1-O15	2.308(9)	Dy2-O10a	2.291(11)	Dy3-O11	2.328(11)
Dy1-O16	2.406(9)	Dy2-O14a	2.499(9)	Dy3-O13	2.420(9)
Dy1-N1	2.517(12)	Dy2-O15a	2.265(10)	Dy3-O16	2.390(9)
Dy1-O14a	2.319(9)				
Bond Angle (o)					
O1-Dy1-O6	52.4(4)	O8-Dy2-O9	78.9(4)	O6-Dy3-O13	77.2(4)
O14-Dy1-N1	112.9(4)	O8-Dy2-O13	110.2(4)	O6-Dy3-O16	75.2(4)
O1-Dy1-O12	78.6(4)	O8-Dy2-O16	75.4(3)	O7-Dy3-O11	73.5(5)
O14-Dy1-O14a	38.2(3)	O5a-Dy2-O8	69.7(4)	O7-Dy3-O13	135.4(4)
O1-Dy1-O13	100.9(4)	O8-Dy2-O10a	143.0(4)	O7-Dy3-O16	84.4(4)
O15-Dy1-O16	150.8(3)	O8-Dy2-O14a	141.6(3)	O11-Dy3-O13	119.7(4)
O1-Dy1-O14	148.3(4)	O8-Dy2-O15a	89.4(4)	O11-Dy3-O16	78.4(4)
O15-Dy1-N1	70.2(4)	O9-Dy2-O13	80.6(3)	O13-Dy3-O16	60.4(3)
O1-Dy1-O15	81.9(4)	O9-Dy2-O16	83.4(4)	O2-Dy3-O3	73.5(4)
O14a-Dy1-O15	83.5(3)	O5a-Dy2-O9	77.2(4)	O2-Dy3-O4	77.5(4)
O1-Dy1-O16	123.2(4)	O9-Dy2-O10a	144.3(4)	O2-Dy3-O6	76.8(4)
O16-Dy1-N1	127.2(3)	O9-Dy2-O14a	137.6(3)	O2-Dy3 -O7	126.2(5)
O1-Dy1-N1	74.4(4)	O9-Dy2-O15a	138.7(4)	O2-Dy3-O11	148.3(4)
O14a-Dy1-O16	76.6(3)	O13-Dy2-O16	60.9(3)	O2-Dy3-O13	66.5(3)
O1-Dy1-O14a	156.6(4)	O5a-Dy2-O13	157.4(3)	O2-Dy3-O16	123.9(3)
O14a-Dy1-N1	83.6(4)	O10a-Dy2-O13	94.4(3)	O3-Dy3-O6	103.5(4)
O6-Dy1-O12	76.8(4)	O13-Dy2-O15a	140.0(3)	O3-Dy3-O7	77.5(5)
O6-Dy1-O13	73.2(3)	O5a-Dy2-O16	136.4(3)	O3-Dy3-O11	89.8(4)
O6-Dy1-O14	141.9(3)	O10a-Dy2-O16	141.6(3)	O3-Dy3-O13	138.8(4)
O6-Dy1-O15	132.6(4)	O14a-Dy2-O16	74.0(3)	O3-Dy3-O16	160.6(4)
O6-Dy1-O16	70.8(3)	O15a-Dy2-O16	93.2(4)	O4-Dy3-O6	148.5(4)
O6-Dy1-N1	103.6(4)	O5a-Dy2-O10a	77.0(4)	O4-Dy3-O7	144.1(4)
O6-Dy1-O14a	143.7(3)	O5a-Dy2-O14a	121.3(3)	O4-Dy3-O11	74.7(4)
O12-Dy1-O13	141.4(3)	O5a-Dy2-O15a	61.6(4)	O4-Dy3-O13	76.0(3)
O12-Dy1-O14	79.8(3)	O10a-Dy2-O15a	88.9(4)	O3-Dy3-O4	86.3(4)
O12-Dy1-O15	121.1(4)	O10a-Dy2-O14a	70.8(3)	O4-Dy3-O16	105.0(4)
O12-Dy1-O16	87.2(3)	O14a-Dy2-O15a	69.8(3)	O6-Dy3-O7	67.3(4)
O12-Dy1-N1	78.2(4)	O13-Dy2-O14a	73.7(3)	O6-Dy3-O11	134.2(4)
O12-Dy1-O14a	117.6(3)	O13-Dy1-O15	135.1(3)	O13-Dy1-N1	67.7(3)
O13-Dy1-O14	110.5(3)	O13-Dy1-O16	60.4(3)	O13-Dy1-O14a	77.1(3)
O14-Dy1-O15	72.7(3)	O14-Dy1-O16	78.5(3)	Dy1-O15-Dy2a	110.5(4)
Dy1-O6-Dy3	94.7(4)	Dy1-O14-Dy1a	141.8(4)	Dy2-O16-Dy3	97.2(3)
Dy1-O13-Dy2	95.3(3)	Dy1-O14-Dy2a	102.9(3)	Dy1-O16-Dy2	96.7(3)
Dy1-O13-Dy3	97.9(3)	Dy1a-O14-Dy2a	95.3(3)	Dy1-O16-Dy3	98.8(3)
Dy2-O13-Dy3	95.0(3)				
